# P-1900. Temporal Course and Risk Factors of Coinfections in Patients Hospitalized for COVID-19

**DOI:** 10.1093/ofid/ofae631.2061

**Published:** 2025-01-29

**Authors:** James L Vilkas, Ali Akmal, Akmal Sarwar, Yuxiu Lei, Timothy N Liesching

**Affiliations:** Lahey Hospital and Medical Center, Burlington, Massachusetts; Lahey Hospital and Medical Center, Burlington, Massachusetts; Lahey Hospital and Medical Center, Burlington, Massachusetts; Lahey Hospital and Medical Center, Burlington, Massachusetts; Lahey Hospital and Medical Center, Burlington, Massachusetts

## Abstract

**Background:**

Studies about the distinction between coinfections acquired prior to admission and hospital-acquired coinfections and risk factors at different time point are limited.

**Figure:**
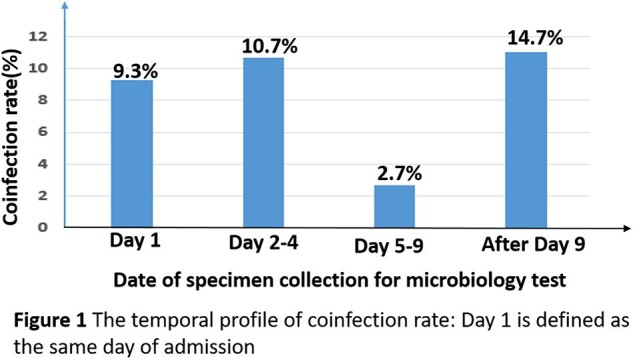

**Methods:**

We reviewed microbiology laboratory data of patients hospitalized for COVID-19 viral infection during January 1, 2024 to March 13, 2024.

**Figure:**
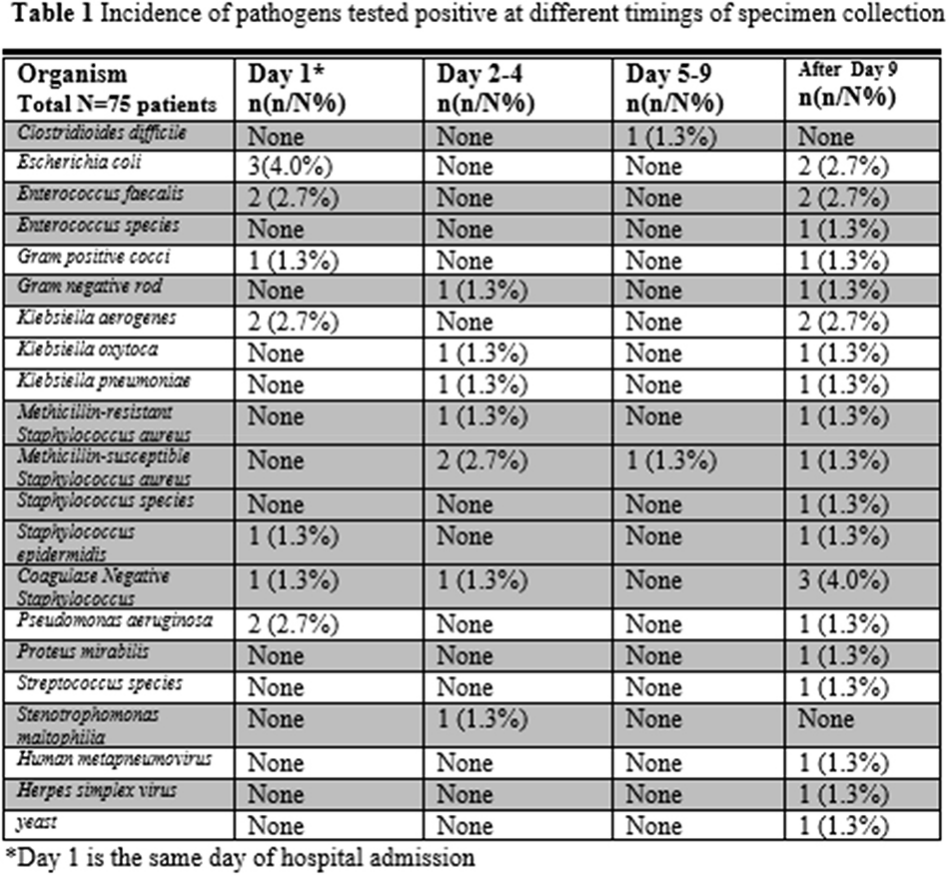

**Results:**

We collected data on 75 patients. The temporal course of coinfection rate was: 9.3% on the same day of hospital admission (Day 1); 10.7% on Day 2 to Day 4; 2.7% on Day 5 to Day 9; 14.7% after Day 9 . At any time point prior to or during the hospitalization or rehospitalization, 23 (30.7%) patients acquired infection of at least one organism.

Univariate analysis showed younger age (69 years vs. 78 years, p=0.025), lower proportion of diabetic patients (17.4% vs. 44.2%, p=0.036), lower proportion of chronic kidney disease (17.4% vs. 40.4%, p=0.065), higher proportion of indwelling catheter (17.4% vs. 1.9%, p=0.029), higher proportion of cancer (34.8% vs.13.5%, p=0.057), similar prior antibiotics (43.5% vs. 36.5%, p=0.6), and significant higher proportion of prior corticosteroids (21.7% vs. 3.9%, p=0.025) in coinfected group than not-coinfected group. Multivariate analysis indicated that indwelling catheter at baseline (OR=45.8, 95%CI: 3.2-652.8, p=0.005) and prior corticosteroids (OR=17.1, 95%CI: 0.99-296.4, p=0.051) are the two independent risk factors for coinfection at admission. Younger age (OR=0.96, 95%CI: 0.918-1.000, p=0.0495) and prior corticosteroids (OR=52.6, 95%CI: 3.11-891.3, p=0.006) are the two independent risk factors for hospital-acquired coinfection.

**Figure:**
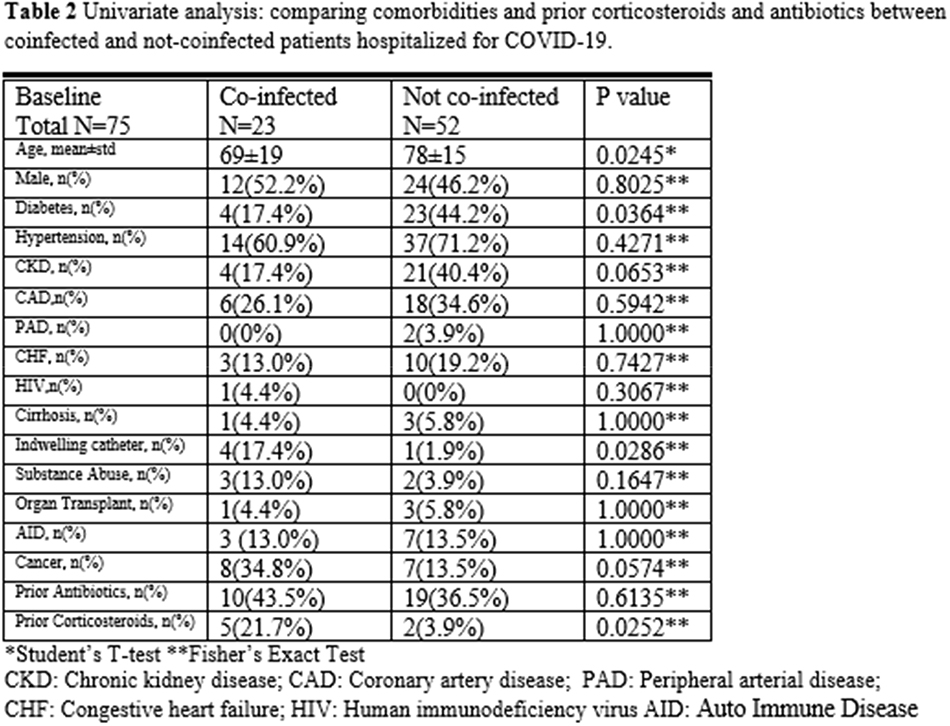

**Conclusion:**

About 10% of patients hospitalized for COVID-19 infection had coinfection(s) at admission, with *Escherichia coli* being the most common. The coinfections at admission were eradicated within eight days. The second wave of coinfection(s) acquired during hospitalization rose to about 15% after eight days, with *Klebsiella* being the most common. Patients with indwelling catheter or prior corticosteroids use are in much greater risk to be coinfected with COVID-19 before hospital admission. Patients with prior corticosteroids are at fifty-time higher risk for hospital-acquired coinfections.

**Figure:**
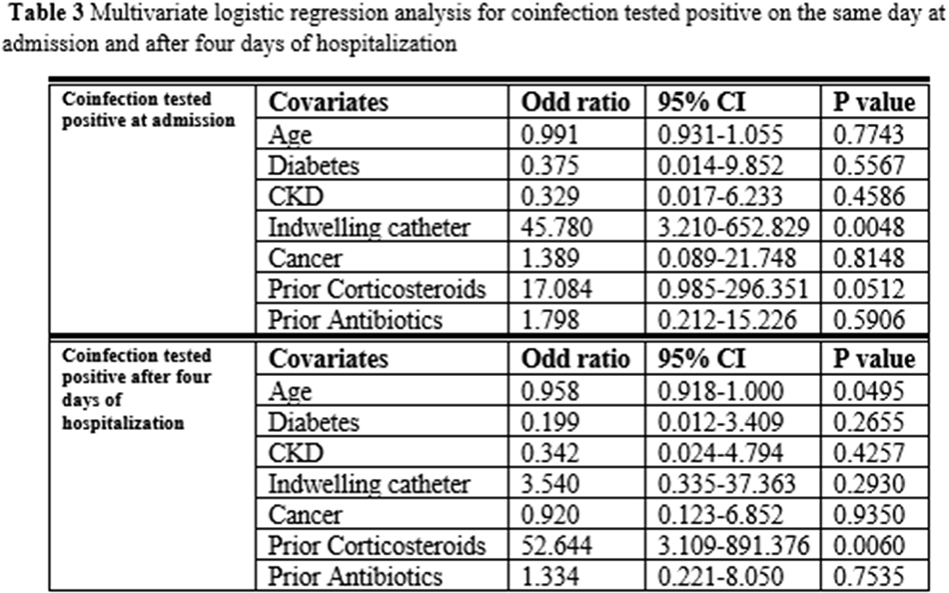

**Disclosures:**

All Authors: No reported disclosures

